# Stakeholder perspectives on scaling up potassium-enriched salt to reduce cardiovascular disease in Australia: a qualitative study

**DOI:** 10.1186/s12889-025-23717-w

**Published:** 2025-07-22

**Authors:** Juliette Crowther, Annet C. Hoek, Kathy Trieu, Inez Denham, Irene Deltetto, Alain Balaguer-Mercado, James D. Bullen, Katrina Kissock, Dori Patay, Emalie Rosewarne, Simone Pettigrew, Bruce Neal, Jacqui Webster

**Affiliations:** 1 The George Institute for Global Health, University of New South Wales, Sydney, Australia; 2 Health Technology Analysts Pty Ltd, Surry Hills, Australia; 3 Business School, University of New South Wales, Sydney, Australia; 4 Leeder Centre for Health Policy, Economics and Data, Charles Perkins Centre, School of Public Health, The University of Sydney, Sydney, Australia; 5 Nutrition, Dietetics and Food Innovation, The University of New South Wales, Sydney, Australia; 6 School of Public Health, University of Technology Sydney, Sydney, Australia

**Keywords:** Potassium-enriched salt, Sodium reduction, Public health intervention, Cardiovascular disease, Hypertension, Food policy, Salt replacement, Industry perspective, Government perspective, Civil society perspective

## Abstract

**Background:**

Cardiovascular disease, the world’s leading cause of death, could be significantly reduced through sodium reduction strategies; however, the implementation of such strategies has had limited impact in Australia and globally. Switching to potassium-enriched salt is a highly promising intervention, but uptake by the food industry and consumers remains limited. This study investigated the barriers and enablers for scaling up potassium-enriched salt use in Australia.

**Methods:**

A qualitative, theory-informed study design was used to conduct 24 semi-structured interviews with representatives from civil society, government, and industry. Interviewees discussed scaling up potassium-enriched salt in relation to their interests, ideas, existing policies and guidelines, and perceived challenges and opportunities within the Australian context. Data were analysed using thematic analysis.

**Results:**

Minimal knowledge and awareness of potassium-enriched salt among all stakeholder groups was the most prominent finding. The key perceived barriers were low consumer demand for potassium-enriched salt products and little incentive for industry to invest in supply. Further, government stakeholders expressed hesitancy to implement policies due to perceived health risks such as hyperkalaemia. Interviewees identified increased awareness, support for industry research and development, and leveraging current policies and initiatives (such as the Australian Health Star Rating system) as potential enablers.

**Conclusion:**

Improving stakeholder understanding of the benefit of switching to potassium-enriched salt in Australia may require a coordinated advocacy strategy that disseminates the evidence and addresses misconceptions. Efforts to drive increased supply and demand could be advanced using a multi-sectoral approach that focuses on supporting industry uptake, encouraging consumer demand, and informing policy implementation.

**Supplementary Information:**

The online version contains supplementary material available at 10.1186/s12889-025-23717-w.

## Background

High blood pressure remains the primary risk factor for cardiovascular disease (CVD) [1], the world’s leading cause of death [2]. Public health strategies to decrease sodium intake and increase potassium intake, such as reformulation initiatives, have been identified as potentially cost-effective ways of reducing blood pressure related disease [3, 4]. Global efforts to lower population sodium intake, including public awareness campaigns, nutrition labelling, and voluntary reformulation, have had limited impact as it has proven difficult to modify the behaviours of consumers and motivate meaningful change by industry in the absence of government legislation [5, 6].

Sodium intake levels in Australia are estimated to be almost double the World Health Organization (WHO) recommendation of < 2000 mg/day for adults [7]. Despite the Australian government signing up to the global target to reduce population sodium intake by 30% by 2030, Australian sodium reduction policies remain weak [8]. With three-quarters of dietary sodium coming from processed foods, Australian government efforts have focused on reformulation and labelling strategies including the Healthy Food Partnership [9], and the Australian Health Star Rating system [10]. To date, these voluntary initiatives have delivered poor results [11, 12]. To reduce cardiovascular risk and meet sodium reduction targets, Australia urgently needs to adopt novel strategies and policies focused on improving the food supply.

Switching to potassium-enriched salt, where a portion of sodium chloride is replaced with potassium chloride, has emerged as a promising scalable strategy to help meet the sodium reduction target and significantly reduce the burden of CVD [5]. Switching regular salt for potassium-enriched salt both in households and food processing may be easier for consumers and industry compared with previous failed efforts to cut out salt [13]. Furthermore, sodium reduction and potassium increase together have a greater blood pressure lowering effect than either one in isolation [14]. A recent major clinical trial in China demonstrated the significant effects of potassium-enriched salt on reducing stroke by 14%, CVD by 13%, and total death by 12% [15]. Further, a systematic review of 21 trials found the blood pressure mediated protective effects of potassium-enriched salt were consistent across different geographies and populations and considered generalisable to countries worldwide [16].

Getting potassium-enriched salt used at scale within a population is likely to be challenging [5]. Food systems are complex due to the vast array of sectors and actors involved, as well as the myriad interacting processes. These processes range from individual food choices, influenced by price, availability, labelling, and preferences, to global market conditions such as global reserves, production, procurement, and trade prices, and global-scale events such as wars and pandemics [17]. Achieving effective scale-up of potassium-enriched salt requires an understanding of the competing interests of different sectors and actors, and developing solutions that address their differing needs [15].

An in-depth review of universal salt iodisation identified that switching the global salt supply to potassium-enriched salt would require a coordinated multisectoral effort with strong industry and government engagement [5]. It also highlighted the need for strong advocacy and communication strategies targeting consumers, food industry, government, health professionals, and academia [5]. Singapore, which is currently at the forefront of promoting potassium-enriched salt as a method to reduce sodium intake, is leveraging multisectoral collaboration to achieve this goal [18]. Government organisations have been engaging with the community, food service sectors, and salt suppliers to encourage increased uptake and supply of potassium-enriched salt in the food industry [18].

While international approaches provide insights that can be used to inform an Australian strategy, each country requires an approach tailored to national food systems and based on a good understanding of local stakeholder positions [5]. Stakeholder perspectives on the use of potassium-enriched salt have been investigated in China and India [19–21], however no studies have explored these perspectives in Australia. Therefore, this study aims to understand stakeholder perspectives on the barriers and enablers to increasing uptake of potassium-enriched salt with a view to scaling up this promising intervention in Australia.

## Methods

Semi-structured interviews with representatives from civil society, government, and industry were used to understand stakeholder perceptions on scaling up the use of potassium-enriched salt. Ethical approval for the study was granted by The University of New South Wales Sydney Human Research Ethics Committee (HC220831).

### Participants and recruitment

Individuals with a role in or understanding of the scale-up of potassium-enriched salt in Australia were recruited using purposeful selection. The study aimed to recruit key informants representing the following sectors: Government – including federal and state government departments (e.g. ministers or senior public servants), statutory and regulatory bodies, or parliamentarians (legislators); Markets (food industry) – including salt and potassium-enriched salt manufacturers, food ingredient manufacturers, food manufacturers, distributors and retailers, hospitality, and other food service venues; and Communities – including consumer groups, civil society and non-government organisations, and academic or research institutions. Participants were eligible for inclusion in the study if they were aware of potassium-enriched salt and if they or their organisation was currently or previously involved in sodium reduction, replacement of salt in the food supply, or food reformulation more generally. Participants were not eligible if they or their company/organisation was not based in Australia.

Potential study participants were identified from existing networks of key contacts and online from organisational websites. This was supplemented with direct referrals from invitees. The snowballing identification of key informants continued until data saturation was reached. It was estimated that this would occur at 10–15 participants for each group, however saturation was reached much earlier (12 industry, 6 civil society and 6 government stakeholders) due to overlapping themes emerging from civil society and government stakeholder interviews. Potential participants were formally invited via email or phone. Those expressing interest were then sent more information regarding the study through a Participant Information Sheet and Consent Form (PISCF). Prior to any data collection, participants were informed about the study and asked if they had any questions before the start of the interview. All participants provided their written or verbal consent to participate and for the interview to be recorded prior to any data collection.

### Data collection

An interview outline was developed to guide the semi-structured interviews. The outline was developed based on the analytical framework and interview guide previously used by one of the researchers (D.P) in non-communicable disease policy research [22] (Supplementary file 1). It covered stakeholders’ priorities and ideas relating to potassium-enriched salt; current policies, regulations, and guidelines; and engagement and strategy options that might influence potassium-enriched salt uptake. From the interview outline, semi-structured interview guides were developed that were tailored to each stakeholder group. For example, government stakeholders were asked about potassium-enriched salt in relation to food policy regulation in Australia, whereas industry stakeholders were asked about sodium reduction reformulation strategies. Examples of how questions were adapted as needed for each stakeholder to best fit their roles included asking industry stakeholders “Does your company see an opportunity to increase/introduce the use of reduced-sodium potassium-enriched salt?” compared with asking government and civil society stakeholders “Would your organisation support the uptake of reduced-sodium potassium-enriched salt in Australia?”.

Interviews were conducted via Microsoft Teams between March and July 2023. Most interviews were between 30 and 40 min in duration and with one respondent (in one instance two people were interviewed who were performing a similar role). Interviews were conducted primarily by two researchers (A.B and In.D) with at least one of two researchers (In.D or Ir.D) sitting in on all interviews to ensure consistency. All interviews were digitally audio recorded and auto transcribed through Microsoft Teams. Audio recordings were listened to by J.C and corrections to the auto transcripts made where necessary and participant identifiers removed.

### Data analysis

Thematic analysis was used to analyse the data [23]. The qualitative research software Dovetail [24] was used to code the data using deductive and inductive approaches concurrently to identify predefined and emerging themes. Initial coding was conducted by one team member (J.C). A second team member (A.H) independently reviewed 5 transcripts (20% of the data) to enhance the validity of the analysis. Regular meetings were held to discuss emerging themes and resolve coding differences. Thematic summaries were drafted and refined to establish the most prominent themes across the stakeholder groups.

## Results

### Participants

In total, 66 Stakeholders were invited to participate in the research. We received a response from 47, and 24 agreed to be interviewed and recorded. The main reason for declining to participate was lack of knowledge/awareness about potassium-enriched salt or lack of authority to represent the organisation’s viewpoint. Interviewees included six civil society stakeholders from consumer or health organisations, six government stakeholders from state or federal health departments, and 12 industry stakeholders, including salt manufacturers, food manufacturers, and members of trade associations. Industry representatives from one Australian food wholesaler and one global fast-food outlet were also interviewed, but did not agree to be recorded or quoted. Written notes were kept of these meetings, and the key perspectives included in the major themes that were identified in the thematic analysis.

### Overview

**Fig. 1:**
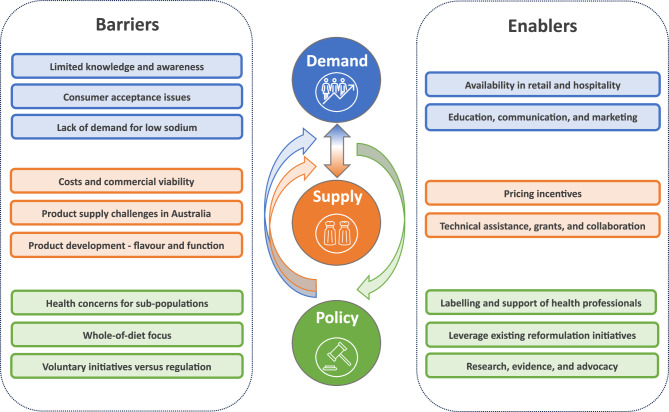
Summary of the key barriers and enablers to scaling up potassium-enriched salt identified by civil society, government, and industry stakeholders

Several common themes were identified within and across the different stakeholder groups. Across all stakeholder groups, most interviewees acknowledged low consumer demand largely arising from limited consumer awareness of potassium-enriched salt. Industry stakeholders were additionally focused on supply issues including costs and commercial viability. Civil society and government perspectives were highly aligned and tended to focus on policy issues such as guidelines moving to a whole-of-diet focus, which discourages a focus on individual nutrients. All interviews highlighted perceived barriers, and although enablers were raised, they were typically not as elaborated upon. A summary of the key barriers and enablers to scaling up potassium-enriched salt identified by all three stakeholder groups is presented in Fig. 1.

### Demand-related barriers

#### Limited knowledge and awareness of potassium-enriched salt

Interviewees from all three stakeholder groups frequently expressed that it was unlikely many consumers would be aware of potassium-enriched salt. They highlighted that consumers would not trust the health benefits of an unknown product. Civil society and government stakeholders noted that it was hard enough to inform consumers about sodium reduction and that potassium-enriched salt would further complicate the issue.

*Anytime we talk to consumers*,* they’re just not aware (potassium-enriched salt) is available. And when you do talk to them*,* they think*,* well it must be chemically engineered. How are you getting the salt out of salt? (Salt supplier-01)*

*People struggle to understand how much sodium they should be having in their food […] Now we’re adding potassium into it. I think it’s going to make it even more difficult for people generally to understand what they’re eating. (Civil-03)*

Many of the stakeholders themselves confessed to having only some awareness of potassium-enriched salt and minimal knowledge about its health benefits or use in Australia. As such, many of the discussions focused more broadly on sodium reduction initiatives that would support potassium-enriched salt uptake. Across all the stakeholder groups, there was agreement that potassium-enriched salt should form part of broader sodium reduction initiatives.

#### Consumer acceptance issues – demand for clean labels

Limited consumer acceptance of a product that may be perceived to be altered was highlighted by members from all three stakeholder groups. Industry stakeholders described an increasing demand for foods with ‘clean labels’ that contain recognisable ingredients.

*The market is moving towards clean labels so people will not want to see something like potassium chloride on their label on the ingredients list. (Industry-01)*

#### Lack of demand for low-sodium

Most interviewees across all three stakeholder groups highlighted a lack of consumer demand for lower-sodium products. Flavour was described as a primary driver for food choice, and consumers were perceived to have an acquired taste for high-sodium products.

*You just have to go and look at the [health brand] range and you’ll see low sodium time and time again […] but they’re not the best sellers. What does that tell you about consumer preference? […] In marketing*,* taste is key. The major consumer doesn’t care about nutrition. They’re not looking for low sodium. (Industry-04)*

Previous failed industry salt reduction efforts leaving consumers with the perception that low salt tastes bad was described by members from all stakeholder groups. Many Industry interviewees spoke of not advertising sodium reduction to avoid losing customers.

*If you tell the consumer anything’s changed*,* they’ll use their imagination to whatever it may be. So we certainly would not advertise the fact that it’s sodium-reduced. (Industry-07)*

### Demand-related enablers

#### Availability in retail and hospitality

Many of the industry interviewees highlighted that amidst the current demand for healthier products, retailers have an opportunity to influence both manufacturers and consumers by choosing to range and promote products containing potassium-enriched salt. They also suggested that with consumers eating more takeaway food, the focus should be on increasing the use of potassium-enriched salt in hospitality.

#### Education, communication, and marketing

Consumer education was viewed as essential by all three stakeholder groups, as well as a need to educate food industry stakeholders on the benefits of potassium-enriched salt. Collaborative education strategies with reputable health and research organisations were considered useful. Industry stakeholders suggested using social media, niche markets, and communication strategies to increase demand.

### Supply-related barriers

#### Commercial interests drive industry action

Civil society and government stakeholders said food industry action was key, but companies were unlikely to act voluntarily as they prioritise profits over public health. Industry stakeholders spoke of conflicting internal company interests with nutrition teams keen to reduce sodium, whilst marketing teams were concerned about losing any commercial benefits.

*The cynical side of health is industry will always do what’s in their interest to do financially […] And there’s many things they can justify on that basis which are also of limited questionable benefit to health. (Government-03)*

*I think they work out ways to make themselves look good without losing their sales. (Civil-04)*

#### Industry costs and commercial viability

The lack of clear commercial benefit and the higher cost of potassium-enriched salt was frequently raised by industry stakeholders as a key reason why companies were hesitant to invest in product changes. They described the wasted investment in product development and the risk of consumer dissatisfaction and brand erosion if reformulated products lack the desired taste or function. Both industry and government stakeholders recognised that smaller businesses face greater financial risks. One industry stakeholder noted it was more profitable to create new products rather than make existing products healthier.

*The investment of going through all these hurdles of a new line*,* you would need to justify it by a proper demand. (Industry-01)*

*It depends on the company in terms of its ability and capability to put resources into sodium reduction […] if you’ve got a company that’s not profitable*,* they don’t have any time or space or money to do anything other than produce food. (Industry-09)*

#### Product supply challenges

Industry stakeholders highlighted that limited global potash mines, importation challenges including potential delays, and significantly higher purchasing costs than regular salt were limiting the use of potassium-enriched salt as an ingredient in food manufacturing. Industry stakeholders said retailers had a significant influence on product manufacturing but attributed the lack of retail availability of potassium-enriched salt products to retailers prioritising products with higher turnover. Some government stakeholders highlighted the limited retail availability of potassium-enriched salt products in regional and remote areas of Australia.

*If the regular salt*,* they can move say six units per store per week and reduced-sodium salt you only can move two units per store per week […] They’re willing to take less margin [from regular salt] because they’ve got higher turnover. (Salt supplier-02)*

#### Product development challenges – flavour and function

The undesirable ‘bitter’ and ‘metallic’ flavour of potassium chloride was frequently highlighted by industry stakeholders as a major barrier with different thresholds for different products/people described. Gradual sodium reduction to adjust consumer taste perceptions was seen by some companies as preferable to a sudden switch in flavour by using potassium-enriched salt, although this was considered time-consuming and costly.

*Depending on your consumer*,* for some people*,* potassium is almost a bitter flavour at some point*,* it starts to give off a bitter note. Different consumers have different sensitivities. (Industry-08)*

The additional functionalities of sodium and limited knowledge of whether potassium-enriched salt would provide the same functionalities, including preservation and safety, providing ‘crispness’ and holding biscuits together was also raised by interviewees from all stakeholder groups.

*If you go down to a certain [sodium] level*,* you’ll lose a certain functionality or you’ll lose your preservation effect or your crispness of tactility or other stuff. So*,* I think there’s a misconception that salt is just in there for flavour enhancement. (Industry-07)*

### Supply-related enablers

#### Pricing incentives

Due to the higher cost of potassium-enriched salt and consumer and industry aversion to product price increases, members from all three stakeholder groups highlighted that pricing incentives may be needed to improve the uptake of potassium-enriched salt by food manufacturers and consumers.

#### Technical assistance, grants, and collaboration

Industry stakeholders identified that research and development grants and partnerships with food technology experts could assist with product development costs and capabilities. Technical assistance and support for smaller companies that lack the resources to conduct their own research was proposed by interviewees from all three stakeholder groups. Potassium-enriched salt manufacturers said the government should subsidise potassium-enriched salt considering the health cost savings that could be gained. Civil society and government stakeholders highlighted that early consultation and collaboration across sectors would be beneficial.

### Policy-related barriers

#### Health concerns for sub-populations

Most civil society and government interviewees described their organisations as being committed to lowering population sodium intakes, given the health benefits. However, many were hesitant about using potassium-enriched salt due to perceived health risks in a small portion of the population, including those at risk of hyperkalaemia. One government stakeholder did note that the risk of hyperkalaemia identified in the Healthy Food Partnership risk assessment was deemed too low to warrant monitoring. There was also concern for those living in remote and regional communities, including Aboriginal and Torres Strait Islander people, due to higher prevalence rates of chronic kidney disease (CKD) in these communities as well as food security issues in remote regions of Australia.

*There needs to be really strong consultation with people*,* particularly in the renal space servicing Aboriginal and Torres Strait Islander communities where we’ve got some of the highest rates of end-stage kidney disease in the world. (Civil-04)*

#### Whole-of-diet focus

Civil society and government stakeholders emphasised that dietary guidelines are moving away from focusing on individual nutrients toward a whole-of-diet focus. Most indicated that while their organisations broadly supported sodium reduction efforts, they did not have sodium-specific guidelines, but rather attempted to reduce sodium consumption using positive messaging about increasing whole foods, such as fruit and vegetables.

*We’re focusing on positive messaging and positive communication. Moving away from that*,* ‘don’t do this*,* don’t eat that nutrient’ focus to an overall heart-healthy eating pattern*,* and part of that is to focus on adding flavour to foods through herbs and spices. So again*,* we’re not saying reduce salt*,* it’s a slightly different way of communicating that. (Civil-01)*

Both civil society and government stakeholders were concerned about potassium-enriched salt eliciting a health halo effect on discretionary food. They were concerned about taking out one ingredient and replacing it with potentially less healthy ingredients and generally voiced a preference for sodium reduction over sodium replacement to reduce consumer preference for salty foods.

#### Voluntary initiatives versus regulation

There was strong support by civil society and government stakeholders to mandate sodium reduction initiatives, with the weak and voluntary nature of existing reformulation efforts cited as a major limitation. Stakeholders described competitive pressure and resource challenges for small businesses as limiting participation. One stakeholder explained that low participation prevents sodium targets from being made stricter for the risk of companies dropping out of the programs.

*If [sodium] targets became mandatory*,* it just becomes so much easier for the consumer because then the reduced sodium options are just what is available in supermarkets. It doesn’t become a matter of choice. Just naturally the diet tends to have less sodium in it. (Government-02)*

There were concerns by some civil society and government stakeholders that mandating potassium-enriched salt use could adversely impact existing salt iodisation initiatives or be seen as a barrier to trade. One government stakeholder indicated that there would likely be a strong pushback from industry on sodium mandates given the current relative success in this area of reformulation. Some stakeholders raised that regulation impacts consumer choice, however, two government stakeholders noted that regulation provides greater equity.

*How will it impact on iodisation*,* if at all? Because that’s an extremely important public health measure that we’ve got and we don’t want to undermine it in any particular way. (Government-03)*

Some government stakeholders described the complexity of the current regulatory processes in Australia as a barrier. They said regulatory change must come from government ministers. However, one government stakeholder described government as tending to be reactive rather than proactive when implementing regulations. Limited staffing and resources, and a lack of coordination between departments, were perceived by government stakeholders to be contributing factors.

*Just from a resource point of view*,* we tend to be a little bit reactive in this space […] teams are reasonably small*,* so we tend to wait for those things [promoting the use of potassium-enriched salt] to bubble up through the system. (Government-06)*

### Policy-related enablers

#### Potassium labelling and support of health professionals

Many civil society and government interviewees strongly favoured mandating potassium labelling to assist consumers at higher risk of hyperkalaemia in making informed choices. They also suggested engaging the support of health professionals to ensure that those who may be at risk of hyperkalaemia are properly informed and monitored.

#### Leverage existing reformulation initiatives

Most civil society and government stakeholders supported mandating existing reformulation and labelling initiatives (e.g. Healthy Food Partnership and Health Star Rating), whereas industry stakeholders spoke of a need for better incentives for more companies to sign up or conform to them. Leveraging environmental, social, and governance (ESG) guidelines was frequently proposed as a viable way to encourage companies to reduce sodium in their products.

#### Research, evidence, and advocacy

Population modelling demonstrating health and economic benefits, along with risk management data on potential unintended consequences, was considered essential for policy implementation by government stakeholders. Civil society stakeholders recommended researchers collaborating with health organisations to advocate to the government a unified message about the health and economic benefits of potassium-enriched salt.

## Discussion

This study of Australian stakeholder perspectives identified limited awareness of potassium-enriched salt across all three stakeholder groups. Industry stakeholders saw minimal commercial gain in using potassium-enriched salt, whereas civil society and government stakeholders were concerned about potential negative health impacts. While several potential actions were proposed to increase uptake, this study identified that an initial focus on improving stakeholder awareness and support for potassium-enriched salt use is likely required to improve the success of future scale-up intervention efforts in Australia.

The existence of a supply-demand stalemate was highlighted in this study. Industry stakeholders described wanting to see higher demand for potassium-enriched salt products before increasing their supply, but limited product availability and promotion, and higher prices for retail potassium-enriched salt, are resulting in low consumer awareness and demand for these products [25]. This is exacerbated by industry stakeholders’ perception that consumers view low sodium unfavourably, are concerned about added ingredients, and are sensitive to product changes, leading to industry using ‘health by stealth’ strategies to reduce sodium, further amplifying a lack of awareness by consumers [20]. Breaking this supply-demand inertia will likely require simultaneous efforts to increase demand while improving the supply and cost of potassium-enriched salt. Parallel strategies by civil society, industry, and government to promote potassium-enriched salt could leverage one another to increase reach and impact. This notably occurred during the United Kingdom’s (UK) salt reduction campaign, where strong government leadership and extensive advocacy led to industry voluntarily reducing sodium, and subsequent parallel efforts by civil society, industry, and government to promote sodium reduction resulted in a measurable shift in consumer behaviours [26].

Industry stakeholders were most concerned about the commercial viability of using potassium-enriched salt, describing the higher procurement and reformulation costs as outweighing the likelihood of commercial gains. Based on median prices globally, potassium-enriched salt is 1.7 times more expensive than regular salt [27]. However, regular salt and potassium-enriched salt are relatively low-cost commodities used in small quantities in food, meaning the costs of switching are not likely to be prohibitive for food manufacturers [27]. Despite this, it seems unlikely, based on the study findings, that they would voluntarily invest in potassium-enriched salt use without significant incentives.

Another key consideration was reformulation challenges, with primary concerns around flavour and function. Potassium chloride has been described as having a bitter or metallic taste [28]. However, several studies have demonstrated that differences between regular salt and potassium-enriched salt are typically undetectable with up to 30% potassium chloride replacement [29–32]. Likewise, potassium-enriched salt has been used in noodles, bread, and pizza without compromising taste [33–36]. Unfavourable taste trials reported by industry may be due to incorrect application of potassium chloride (such as 100% replacement) [30]. The challenges raised in this study have frequently been cited in relation to other sodium reduction and food reformulation initiatives by the food industry [5, 19, 37] and may reflect the greater profitability in making new products, as described by some stakeholders in this study. While positive showcasing of potential commercial benefits, including promoting environmental, social, and governance gains and business opportunities for flavour manufacturers, might encourage industry uptake, it is likely that stronger incentives, in the form of regulation, may be required.

Current indications that the market is not meeting sodium reduction targets through voluntary initiatives highlight a need for stronger action [8, 38, 39]. Stakeholders in this study highlighted that creating a level playing field through regulation would assist in addressing company hesitation to reduce sodium or conform with voluntary guidelines for fear of losing market share. Modelling studies and systematic reviews assessing the effectiveness of previous salt reduction strategies have shown that legislative approaches are more effective by resulting in greater reductions in population salt intake [40–42]. Further, the current policy landscape shifting to a whole-of-diet focus means soft policy actions like promotional campaigns are unlikely to focus on sodium or potassium. As such, regulating the existing targets and initiatives that focus on nutrients (e.g. the Healthy Food Partnership and Health Star Rating system) could be a viable way to ensure the food industry meets reductions.

Civil society and government stakeholders focused more on the potential health risks of potassium-enriched salt rather than the likely population benefits. This finding aligns with previous research in which government stakeholders highlighted safety concerns about potassium-enriched salt, whereas industry and research stakeholders expressed confidence in safety [19]. Patients at risk of hyperkalaemia (high serum potassium levels), such as those with advanced CKD or taking certain medications, such as potassium-sparing diuretics, are advised to avoid potassium [43]. There is limited evidence on the effects of potassium-enriched salt use in these populations due to their exclusion from most clinical trials [16]. However, no harm has been found in any potassium-enriched salt trial to date, including trials in people with potentially undiagnosed CKD and elderly participants at higher risk of hyperkalaemia [16, 44]. Risk-benefit analyses conducted in the UK and China have demonstrated that the population-level benefits of using potassium-enriched salt to reduce sodium far outweigh the potential risks, with benefits projected even amongst those at elevated risk of hyperkalaemia [45, 46]. In the context of this study, stakeholder perspectives may have been influenced by the draft WHO guidelines released in 2023 that recommended ‘limited use of low-sodium salt substitutes’ [47]. Following an extensive review process, the WHO released its final guidelines on lower salt substitutes in January 2025, which support using lower sodium salt substitutes that contain potassium in place of regular table salt among the general adult population [27]. Globally, health organisations increasingly support the inclusion of potassium-enriched salt in clinical guidelines, including in the 2024 European Society of Cardiology guidelines and the 2023 European Society for Hypertension guidelines [48]. Improving stakeholder support will require these guideline updates to be effectively communicated to health organisations and policymakers.

Gaps in understanding around the impact of interventions on potentially vulnerable groups, including Aboriginal and Torres Strait Islander populations, were raised as a concern in this study. Australian Institute of Health and Welfare data indicate that among First Nations People, rates of CKD are more than two times higher, and rates of death caused by CKD are four times higher, compared to non-indigenous Australians [49]. No potassium-enriched salt studies have investigated health impacts in Aboriginal and Torres Strait Islander populations. Confirming the safety of interventions among these populations needs to be a priority area of future research. The promotion of potassium-enriched salt in clinical hypertension management settings, where hyperkalaemia risks can be mitigated through medical management, may serve as a safe early focus of scale-up activities [5, 48].

### Strengths and limitations

Our research methods allowed a thorough assessment of different stakeholder perspectives. While self-selection bias and a modest sample size may have influenced the results, the study was based on a diverse sample of key stakeholders from relevant food and health sectors across most states and territories of Australia. The lack of representation by health professionals and limited input from retail and hospitality sectors was a key limitation, however, the inclusion of other key industry actors including manufacturers and salt suppliers was an important strength of this study. Future research would benefit from ensuring representation from all sectors or by focusing specifically on underrepresented sectors including retail, hospitality, and health professionals. The finding that most stakeholders were not highly knowledgeable about potassium-enriched salt meant the researchers were unable to obtain informed perspectives on technological feasibility or intervention strategies. This study was limited to Australian stakeholders to inform a scale-up strategy in Australia, however many of the findings align with international research [19, 20, 50], and may therefore be insightful for other countries.

### Implications and recommendations

Efforts to increase the uptake of potassium-enriched salt in Australia should focus on improving stakeholder knowledge and awareness of potassium-enriched salt and its potential as a public health tool. Targeted dissemination of the existing evidence could help to break the current supply-demand stalemate described by interviewees. Each sector has a role to play in managing safety concerns, promoting potassium-enriched salt, increasing supply, and reducing costs. Multi-sectoral action on potassium-enriched salt strategies could benefit all sectors through knowledge exchange, decreased costs, improved reach, and beneficial health and economic outcomes [51]. An initial focus on increasing awareness among healthcare professionals and engaging the support of health and civil society organisations could mitigate safety concerns, improve government support and assist with advocacy efforts for effective policies [5]. Future research should focus on addressing safety concerns for at-risk populations. Monitoring and evaluating implemented strategies will also be vital for documenting progress, identifying gaps, and understanding how to enhance strategies for improved outcomes.

## Conclusions

Despite well documented evidence on the health benefits of potassium-enriched salt to reduce cardiovascular disease prevalence, this study identified low stakeholder awareness and support for potassium-enriched salt. Improving stakeholder support for switching to potassium-enriched salt through a coordinated advocacy strategy to disseminate the evidence and address misconceptions will be an important next step. In parallel, supply and demand challenges identified by stakeholders could be addressed through a multi-sectoral approach that focuses on supporting industry uptake, encouraging consumer demand, and informing policy implementation.

## Supplementary Information

Supplementary Material 1.

## Data Availability

The datasets generated and/or analysed during the current study are available from the corresponding author on reasonable request.

## Abbreviations

CVD: Cardiovascular Disease
WHO: World Health Organization
ESG: Environmental, Social and Governance
UK: United Kingdom
CKD: Chronic Kidney Disease
