# Supplementary material for: Stakeholder perspectives on scaling up potassium-enriched salt to reduce cardiovascular disease in Australia: a qualitative study
Source: BMC Public Health. 2025 Jul 22;25:2525. doi: 10.1186/s12889-025-23717-w (PMC12281820; doi:10.1186/s12889-025-23717-w)
Supplement: Supplementary file 1 — Supplementary Material 1. [file 12889_2025_23717_MOESM1_ESM.docx]

**Supplementary File 1. Stakeholder interview outline**

**Stakeholder Perspective:** ‘Scaling-up reduced-sodium salts to reduce cardiovascular disease and stroke: influencing governments, markets and communities’ project

**PARTICIPANT INFORMATION**

| Participant Identifier Number |  |
| --- | --- |
| Date |  |

**BACKGROUND**

Participants will be asked about…

**INTRODUCTION**

Thank you for taking the time to take this interview. The aim of this interview is to discuss…

We want to get your opinions about…

We would also like to remind you that this discussion is being audio-recorded. Any personally identifiable information is removed during transcription, and responses are made anonymous**.**

**INTERVIEW TOPICS**

1. Introduction: opinions and ideas about reduced-sodium salts

Example questions:

- What do you think about reduced-sodium salts?
- Do you think that the uptake of reduced-sodium salts could benefit the health of the Australian population? Why?
- What opportunities and issues do you perceive in increasing their uptake in Australia?

1. Interests and mandates

Example questions:

- Would your organisation support or oppose the uptake of reduced-sodium salts in Australia? Why?
- How would your organisation support/oppose their uptake?
- Which organisations/agencies/companies would support/oppose the uptake of reduced-sodium salts in Australia? Why?

1. Power, influence and social network

Example questions:

- What other organisations/agencies/companies do you talk with on these matters? Do you partner with any of these actors?
- In your opinion, who are the most influential actors in this space who can influence the uptake of reduced-sodium salts?
- Where does the power of these actors come from? (e.g., money, expertise, connections)

1. Regulation

Example questions:

- Are there any regulations/policies that facilitate/constrain the uptake of reduced-sodium salts in Australia?
- What regulation/policies should be put in place/amended to facilitate/constrain the uptake of reduced-sodium salts in Australia?

1. Engagement and strategies

Example questions:

- What would be needed to persuade the food manufacturers/food ingredient manufacturers/government agencies/civil society/consumers to support the uptake of reduced-sodium salts?
- In what ways can food manufacturers/food ingredient manufacturers/government agencies/civil society/consumers engaged to strengthen their support for the uptake of reduced-sodium salts?
